# Timing and awareness of movement decisions: does consciousness really come too late?

**DOI:** 10.3389/fnhum.2013.00385

**Published:** 2013-07-30

**Authors:** Adrian G. Guggisberg, Anaïs Mottaz

**Affiliations:** Division of Neurorehabilitation, Department of Clinical Neurosciences, University Hospital of GenevaGeneva, Switzerland

**Keywords:** volition, Libet, free will, introspection, decision-making

## Abstract

Since Libet's seminal observation that a brain potential related to movement preparation occurs before participants report to be aware of their movement intention, it has been debated whether consciousness has causal influence on movement decisions. Here we review recent advances that provide new insights into the dynamics of human decision-making and question the validity of different markers used for determining the onset of neural and conscious events. Motor decisions involve multiple stages of goal evaluation, intention formation, and action execution. While the validity of the Bereitschaftspotential (BP) as index of neural movement preparation is controversial, improved neural markers are able to predict decision outcome even at early stages. Participants report being conscious of their decisions only at the time of final intention formation, just before the primary motor cortex starts executing the chosen action. However, accumulating evidence suggests that this is an artifact of Libet's clock method used for assessing consciousness. More refined methods suggest that intention consciousness does not appear instantaneously but builds up progressively. In this view, early neural markers of decision outcome are not unconscious but simply reflect conscious goal evaluation stages which are not final yet and therefore not reported with the clock method. Alternatives to the Libet clock are discussed that might allow for assessment of consciousness during decision making with improved sensitivity to early decision stages and with less influence from meta-conscious and perceptual inferences.

## Introduction

We have the strong and intuitive conviction that we can make decisions and actions according to our own reflections, preferences, beliefs, and feelings. In other words, we take it for granted that the content of our consciousness somehow influences our decisions and actions. However, this assumption has been seriously challenged by a seminal study of Benjamin Libet published in the eighties (Libet et al., 1983). Healthy human participants had to make repeated, self-paced finger movements while their brain activity was recorded with EEG. In addition, they were asked to watch a rapidly rotating clock and to report, after each movement, the clock hand position at the moment they had consciously “felt the urge to move.” It was found that a so-called Bereitschaftspotential (BP), a brain potential related to voluntary movements, starts several hundred milliseconds before the participants reported to be conscious about their decision to move. This result, which has been reproduced and extended by independent groups (Haggard and Eimer, 1999; Soon et al., 2008; Fried et al., 2011; Rigoni et al., 2013a), seems to indicate that consciousness about a movement decision arises only after the decision has been made by unconscious neural processes.

The study and its replications have had massive impact on current concepts of volition and continue to be used as one of the main arguments against the existence of a freedom to choose. It seems to demonstrate that our actions are determined by unconscious neural processes whereas consciousness is merely a late byproduct of neural processing with no influence on its own. Interestingly, this conclusion may have negative consequences for our conceptions of freedom and responsibility and hence for the functioning of society. Indeed, studies have demonstrated that healthy volunteers reading texts coming to such conclusions immediately have a greater tendency to cheat (Vohs and Schooler, 2008), are more likely to conform to group norms (Alquist et al., 2013), and show reduced social behavior (Baumeister et al., 2009), weakened self-control (Rigoni et al., 2012), impaired cognitive reactions to errors (Rigoni et al., 2013b), as well as reduced BPs (Rigoni et al., 2011).

However, a growing body of evidence suggests that the relationship between neural activity and conscious awareness is more complicated than previously thought, and other conclusions can be drawn. In this paper, we review the markers that have been used to determine the onset of consciousness on the one hand and the onset of brain processes for movement preparation on the other hand, and consider whether they are valid and adequate.

## Markers of neural decision processes

### The bereitschaftspotential

Libet used the onset of the so-called BP as marker for the onset of neural movement preparation. The BP is a slow, surface negative potential that can be obtained by recording EEG epochs before repeated self-paced movements and averaging them time-locked to movement onset (Kornhuber and Deecke, 1965; Shibasaki et al., 1980). At least 2 different components can be distinguished: the first component starts about 2 s prior to movement onset and is bilateral symmetrically distributed with a maximum over the supplementary motor area (SMA). The second component starts about 0.4 s before movement onset and has an asymmetrical distribution with a maximum recorded above the contralateral primary motor cortex (Shibasaki et al., 1980; Shibasaki and Hallett, 2006).

The usage of the early component of the BP as neural marker of internal movement generation has been justified by the following findings (Shibasaki and Hallett, 2006).

1. The BP was initially thought to occur only before self-paced but not before externally-paced movements (Libet et al., 1982; Papa et al., 1991), but see below. It is also usually absent before pathological and involuntary movements such as periodic leg movements (Trenkwalder et al., 1993), tics (Obeso et al., 1981), or myoclonus (Shibasaki and Kuroiwa, 1975), although there are several exceptions (Karp et al., 1996; Oga et al., 2000).
2. The onset of the BP seems to be earlier for preplanned than for spontaneous movements (Libet et al., 1982).
3. The early component of the BP has greater amplitude when a selection between 2 or more movements is made, compared to self-paced movements without selection (Praamstra et al., 1995; Dirnberger et al., 1998), and it is greater before sequential than before single movements (Kitamura et al., 1993).
4. Intracerebral depth recordings showed that the BP occurs mostly in regions belonging to the sensorimotor system, i.e., the SMA, primary motor and sensory cortices, premotor cortex and the cingulate gyrus, but not in temporal or parietal regions (Shibasaki and Hallett, 2006; Sochurkova et al., 2006). There is a large body of evidence that these regions are implicated in movement preparation (Fried et al., 1991; Thaler et al., 1995; Deiber et al., 1996, 1999; Ball et al., 1999; Zhang et al., 2012).

However, the following arguments suggest that the BP is in fact not an adequate marker for actual movement decisions, but that it represents a diffuse and non-specific preparation of the cortex for future tasks.

1. The BP belongs to the family of direct current (DC) potentials, the origin and generators of which are incompletely understood and a matter of debate. There is evidence for neuronal mechanisms generating DC potentials, including excitatory postsynaptic potentials at apical dendrites due to synaptic input from unspecific thalamic afferences and axonal collaterals (Caspers et al., 1980; Birbaumer et al., 1990), and hyperpolarization of the neuronal membrane following sustained and coordinated spiking of nearby neurons (Buzsaki et al., 2012). In addition, non-neural current sources such as glial cells, extracellular potassium concentrations, and potential differences across epithelia of the blood-brain barrier seem to contribute as least as much to DC potential shifts at the surface (Voipio et al., 2003). Negative shifts in DC potentials can be recorded under many different circumstances including during the transition from sleep to wakefulness, sensory stimulation, attention shifts, hypoxia, and epileptic seizures (Caspers and Schulze, 1959; Caspers et al., 1980; Birbaumer et al., 1990). Given these mechanisms and circumstances, DC potentials likely represent unspecific regional workload, attention and vigilance rather than specific neural computation.
2. Unlike initially thought, DC potentials similar to the BP can also occur before externally cued movements (Thickbroom et al., 1985; Keller and Heckhausen, 1990; Jahanshahi et al., 1995; Herrmann et al., 2008). Furthermore, they can be observed before expected external stimuli without movements (Brunia, 1988) or even before participants decide *not* to move (Trevena and Miller, 2010). In these latter cases, the DC potentials are usually not named BP but *contingent negative variation (CNV)* or *stimulus preceding negativity (SPN)*, due to the different context of occurrence. Nevertheless, they share common mechanisms and configurations (Brunia, 1988). In any case, CNV and SPN demonstrate that expecting a future event is sufficient to trigger DC potentials and hence movements may not be necessary for the generation of BP either.
3. A DC potential similar to the BP also precedes the onset of visual cues instructing the participants to move one of two buttons. Hence, a BP-like potential started at least 600 ms before participants could know when or which hand to move, thus suggesting that is related to task expectation rather than actual movement preparation (Herrmann et al., 2008).
4. Not all self-paced movements are preceded by a BP. Hence, the BP is not necessary for internal movement generation (Pockett and Purdy, 2010).
5. The amplitude and configuration of the BP can superimpose on other DC potentials related to concomitant tasks such as spatial attention or visual processing (Lang et al., 1984), in which case it is impossible to distinguish between motor and non-motor processes. In this regard, the concomitant clock-task used for determination of the onset of consciousness in Libet's experiment is of particular concern, as it may contaminate the onset of the BP. Several studies have found that this is indeed the case: instructing the participants to pay attention to their decisions leads to an earlier onset of the BP (Keller and Heckhausen, 1990; Sirigu et al., 2004; Miller et al., 2011). Hence, the mere fact that the participants in Libet's experiment had to determine the onset of their conscious urge to move seems to have biased the marker used for assessing neural movement preparation.
6. Even random fluctuations in neural activity can produce a potential similar to the BP if the movement decision is based on a threshold crossing of these fluctuations (Schurger et al., 2012).
7. Lesions to the posterior parietal cortex (Sirigu et al., 2004) and the cerebellum (Kitamura et al., 1999) lead to an important reduction or even disappearance of the BP, thus suggesting that it depends not only on the motor system but on very distributed neural networks.
8. The onset of the early component of the BP does not co-vary with the reported time of conscious awareness. This provides evidence against Libet's conclusion that subconscious neural processes underlying the BP are the cause of subsequent consciousness of motor decisions (Haggard and Eimer, 1999).

Hence, although Libet used the best available marker of neural motor processing of his time, its validity is controversial today. Activity in the SMA and primary motor areas contribute to the BP, but it also seems to be heavily influenced by non-specific and even non-neural processes. The meaning of the BP remains therefore unclear. It seems to be related to unspecific attention and task expectation in which case it merely indicates that the study participants prepared for some upcoming task in each trial. In addition, motor-related processed may contribute to its amplitude, but it may be difficult to discriminate between motor-related and unspecific components. Hence, the onset of the BP seems to be an unreliable indicator of the time when the brain starts preparing a movement.

Yet, the question of how and when conscious considerations influence decisions and actions remains important. Several groups have therefore tried to find better markers for investigating neural decision processes, which might enable us to address Libet's original question more appropriately.

### The lateralized readiness potential

The lateralized readiness potential (LRP) is a variant of the BP and a more specific marker for movement preparation (Coles, 1989). It is obtained by subtracting the BP of the central electrode located over the hemisphere of the same side as the moved limb from the BP of the electrode on the opposite side. It therefore measures the extent to which the contralateral motor cortex mediating a movement is more active than the ipsilateral motor cortex. It depends mostly on the late component of the BP which is more directly related to actual movement preparation than the early component (Shibasaki et al., 1980). Hence, although much less information about the specificity and the precise meaning of the LRP is available, it seems to avoid many of the problems of the BP.

Haggard and Eimer (1999) used the LRP in a slight modification of Libet's original paradigm. Rather than just performing repetitive, self-paced movements with one hand, the participants of their study additionally had to choose between left and right hand movement in each trial. This modification allowed them to use the onset of the LRP as the moment at which the brain decided to prepare the movement of the contralateral rather than the ipsilateral hand. This time was then again compared to the time of conscious decisions that the participants reported using the clock paradigm developed by Libet. They observed that participants reported to be conscious about their decisions on average 350 ms before the movements, whereas the LRP started already about 800 ms before the movements. Hence, the participants reported to be conscious about their choice only about 450 ms after the brain had started to prepare the chosen action. This confirms Libet's original finding with a more robust marker of neural motor preparation. Furthermore, the onset times of the LRP only preceded but, unlike the BP, also systematically co-varied with the times of conscious intentions reported by the participants. A recent study assessed DC potentials in the motor cortex with a Laplacian transform of surface EEG recordings and reported similar results (Rigoni et al., 2013a).

### fMRI movement predictors

**Figure 1:**
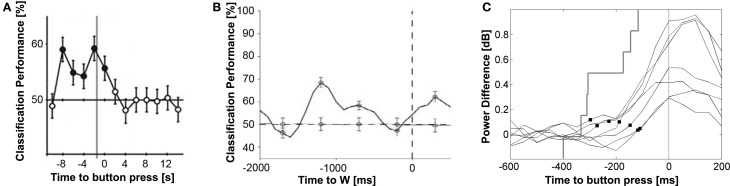
**Examples of neural predictors of movement decisions during two-option forced choice tasks. (A)** The pattern of fMRI activation in the fronto-polar cortex predicts the chosen movement up to 8 s before the button press. The gray vertical line indicates the earliest time point of conscious intentions reported by the participants. Significant time points are marked with filled circles. Adapted from Soon et al. (2008) by permission from Macmillan Publishers Ltd.: *Nature Neuroscience*. **(B)** The neural firing rate of neurons in medial frontal and temporal cortex (solid line) predicts the chosen movement more than 1 s before participants report to be conscious of their intention (dashed vertical line). The dashed horizontal line represents the classifier performance upon shuffling left-right responses. From Fried et al. (2011) with permission from Cell Press. **(C)** High-gamma activity in the primary motor cortices of 7 participants (solid curves) discriminates between left and right movement decisions after participants report their intentions. Black squares mark the onset of an activity difference in the primary motor cortices indicating that the brain starts preparing the chosen movement. The gray stair plot shows the cumulative distribution of subjective intentions reported by the participants. The earliest high-gamma activation differences in M1 between left vs. right hand choices were observed at 194 ms (±74 ms) before movement onset, which was not significantly different from the subjective intention onset reported at 255 ± 109 ms [*t*_(6)_ = 1.1, *p* = 0.32]. Modified from Guggisberg et al. (2011) with permission from Elsevier.

When using sophisticated classifiers to learn and decode the intentions of human participants from fMRI signals, researchers were able to predict, with a small but significant probability of 55–60%, future decisions already up to 7 s before the participants reported to become aware of their decisions and up to 8 s before the actual movements (Figure 1A). The earliest predictions could be made when looking at fMRI activity patterns in the frontopolar cortex. The primary and supplementary motor cortices were also predictive, but only after the earliest times of conscious decisions reported by the participants. The onset of conscious decisions was measured using a variant of Libet's original clock paradigm optimized for fMRI. As in Libet's original study, these findings again suggested that “a network of high-level control areas begins to prepare an upcoming decision long before it enters awareness” (Soon et al., 2008). This time, the predictors of future decisions were validated with state of the art techniques.

### Neural firing rate

Fried et al. (2011) recorded activity of neurons in human participants while they performed self-paced finger movements. They observed a progressive recruitment of neurons in the supplementary motor starting about 1000 ms before participants reported to be conscious about their decisions. Hence, changes in firing rate of individual neurons showed a similar time course as the BP recorded at the surface. They were able to predict the time point of future movements from the firing rate of neurons with a mean error of 152 ms. In addition, the spiking rate of neurons in the medial frontal and temporal lobes significantly predicted the chosen movement in a two-option forced choice task with an accuracy of ~70%, more than 1 s before participants reported to have a conscious movement intention (Figure 1B). The predictive value of firing rate for motor decisions was verified with crossvalidation techniques.

### High-gamma oscillations

Recent studies using intracranial and surface EEG recordings have demonstrated that fast neural oscillations in the so-called gamma and high-gamma frequency range (~40–200 Hz) are reliable and specific markers of local neural processing that outperform traditional EEG/MEG and fMRI markers in combined spatiotemporal resolution.

1. Like slower EEG/MEG rhythms, gamma, and high-gamma oscillations result from postsynaptic currents and therefore reflect synaptic input (Buzsaki et al., 2012). Yet, in contrast to other rhythms, they also correlate with the spiking rate of nearby neurons (Rasch et al., 2008; Whittingstall and Logothetis, 2009). Hence they also contain information about the output of local neural computation.
2. Unlike other EEG/MEG rhythms, they correlate positively with the fMRI hemodynamic response (Logothetis et al., 2001; Brovelli et al., 2005; Niessing et al., 2005). Hence they reflect local neural activity while having much better time resolution than fMRI.
3. They are spatially more focal and more task specific than slow neural oscillations and event-related potentials (Brovelli et al., 2005; Edwards et al., 2005; Crone et al., 2006; Canolty et al., 2007).
4. In intracranial recordings, they have a sufficiently high signal-to-noise ratio to allow tracking even the time course of neural processing in single trials (Edwards et al., 2010).

High-gamma oscillations are therefore excellent indices of neural activity for assessing the dynamics of cortical processing. Advances in source localization algorithms allow reconstructing high-gamma rhythms also from surface recordings, given sufficient repetitions of a task (Dalal et al., 2008). Thus, it is possible to watch the brain decide, i.e., to look into the dynamic neural processes underlying human decision-making. Guggisberg et al. (2011) compared high-gamma markers of neural processing to subjective times of decision onset obtained with the clock method introduced by Libet. It was found that participants report to be conscious about their choice at the time point at which high-gamma activity in the motor cortex contralateral to the moved finger starts to increase more than high-gamma activity in the ipsilateral hemisphere indicating that the brain starts preparing the chosen action. Hence, when using high-gamma activity of the motor cortex as specific marker of cortical movement preparation, there was no evidence for a delayed onset of conscious awareness in forced choice tasks (Figure 1C).

## Neural dynamics of movement decisions

**Figure 2:**
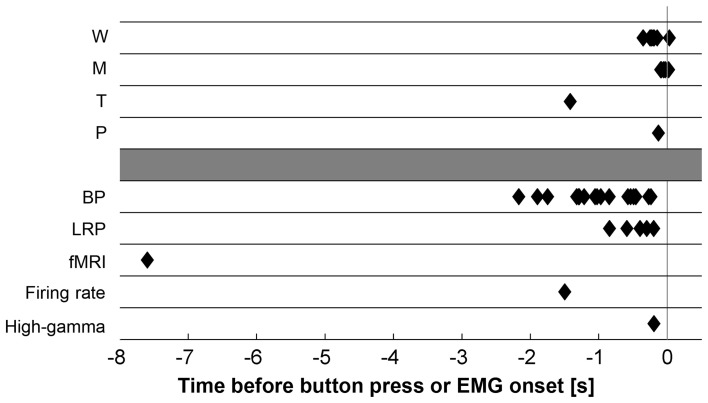
**Summary of subjective (top) and neural (bottom) markers of motor decisions reported in the literature**. W, subjective intention onset as determined with the Libet clock; M, subjective onset of finger movement as determined with the Libet clock; T, subjective intention onset as determined with random sampling; P, point of no return after which participants cannot stop an intended movement anymore; BP, Bereitschaftspotential; LRP, lateralized readiness potential; fMRI, predictors of movement decisions decoded from fMRI recordings; Firing rate, predictors of movement times decoded from neural firing rate in the SMA; High-gamma, significant differences in high-gamma power in the left vs. right primary motor cortex during left vs. right finger movements; EMG, electromyogram.

Figure 2 and Supplementary Tables S1, S2 recapitulate the onset times of neural and subjective events reported in the literature on movement decisions. The different markers yield highly variable results. How can we explain the divergences between the different studies? The following sections will discuss several factors and propose an interpretation.

The highly variable onset times of neural events predicting decisions strongly suggest that the different markers capture different stages in the dynamic process of decision-making. But which are these stages and at which time point in the sequence of neural activations do participants report to be conscious about their intentions?

Several excellent reviews have proposed models on the neural dynamics of motor decisions (Brass and Haggard, 2008; Haggard, 2008; De Jong, 2011; Krieghoff et al., 2011; Brass et al., 2013). Haggard (2008) suggested that human volition involves 4 distinct processing stages.

1. *Early whether* decision: new motivations and reasons for action appear and take over the control of the action system from routine stimulus-based responding.
2. *What* decisions: the person first selects a goal among several options and then the movements necessary to achieve this goal.
3. *Late whether* decisions: a forward model is built based on information about the chosen voluntary action and compared to the initial goal description. This final check may result in adjustments or even in a complete abortion (veto) of the planned movement. It is only at this stage that the definitive choice for an action is made.
4. The chosen action is executed.

This sequence adapts to different choice contexts (Brass and Haggard, 2008; Krieghoff et al., 2011). In the case of simple self-paced *when* decisions as studied in the original Libet paradigm, the *what* decision may be absent whereas the action time receives more attention. This seems to be associated with a particular recruitment of the pre-supplementary motor area (pre-SMA) (Gerloff et al., 1998; Deiber et al., 1999; Jenkins et al., 2000). Guggisberg et al. (2008) localized high-gamma activations from magnetoencephalography (MEG) recordings obtained during two-option forced choice decisions with and without perceptual information. The observed brain activations during this choice context were divided into 4 overlapping processing stages corresponding to 4 cognitive steps of choice.

1. Perception of the options mediated by primary and secondary sensory cortices; e.g., the individual sees two kinds of ice cream.
2. Retrieval of information regarding the value of the options. If the 2 options are perceptually different, the sensory information is analyzed by specialized brain areas; e.g., appearance and price of the options are compared, memories of earlier experiences with the options are recalled, and personal preferences analyzed.
3. Attribution of an overall value to each option and formation of an intention corresponding to the option with the highest current value. This stage seems to involve the parietal cortex and the SMA. If the options are perceptually equal, the inferior parietal lobule is activated.
4. Execution of the action corresponding to the choice, mediated by the motor cortex.

Similar dynamics have also been described in other studies with other neural oscillation frequencies (e.g., Siegel et al., 2011). Free decisions without perceptual discrimination of the value of the options are based on increased recruitment of the bilateral inferior parietal lobules and the cerebellum compared to decisions with perceptual information (Deiber et al., 1996; Guggisberg et al., 2008) although they also share common resources (Bode et al., 2013). More importantly, free decisions can be predicted earlier than value-constrained decisions (Bode et al., 2012), and possibly even from random noise in neuronal spiking (Rolls and Deco, 2011).

Evaluation stages lead to an accumulation of activity in mesial motor areas (in particular the SMA) until it crosses a threshold which finally leads to a release of the selected action (Fried et al., 2011; Zhang et al., 2012).

In sum, the different neural predictors of future decisions reported in the literature likely capture different stages of neural decision processing and are derived from different decision types. For instance, fMRI patterns in the fronto-polar cortex might correspond to *early whether* stages of free *what* decisions. In contrast, high-gamma activity in the primary motor cortex is probably related to late stages of action preparation and execution. This may explain the large differences in the onset times of these markers (Figure 2).

**Figure 3:**
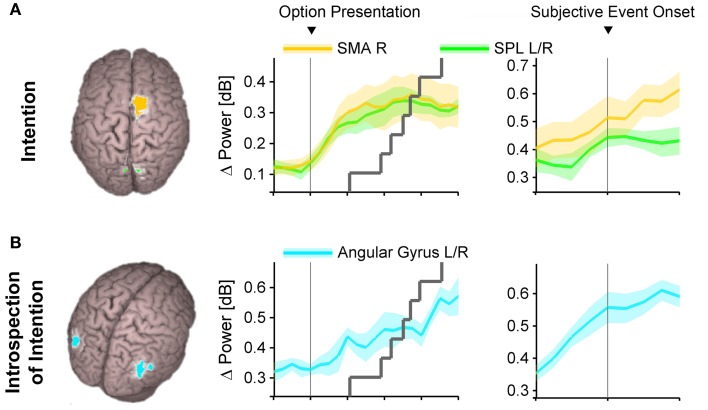
**Spatially distinct neural substrates of primary intention (A) and introspection of intentions (B)**. Abbreviations: SMA, supplementary motor area; SPL, superior parietal lobule; L, left; R, right. Reproduced from Guggisberg et al. (2011) with permission from Elsevier.

To come back to Libet's question, when in the sequence of neural choice processing do humans report to become conscious of their intentions? To address this, Guggisberg et al. (2011) reconstructed high-gamma activations during a forced choice task at the reported subjective time point of conscious intention. They found significant activations in brain regions responsible for the formation of intentions, i.e., in the SMA and the posterior parietal cortex (Figure 3A). This was the case both for free as well as for value-constrained decisions. Hence, participants report the onset of their decisions roughly at the time of high-gamma activity rises in brain areas responsible for intention, just before high-gamma activity in the primary motor cortex indicates the preparation of a chosen movement. This suggests that conscious volition is reported at the *late whether* decision stage, when the final intention is formed and just before the forward model is accepted and released for execution. It is probable that participants report their conscious motor urges at a similar stage of final intention formation also in case of self-paced movements as studied in Libet's original paradigm.

Now, several studies were able to predict future decisions already from neural processes occurring long before this *late whether* stage of decision-making. Participants did not report to be conscious of their decisions during these earlier stages, even though the choices could be predicted from markers of neural activity. Does this mean that decisions are unconsciously generated? Before we can conclude this, we need to verify whether the measures used for determining the onset time of conscious decisions are valid.

## Markers of consciousness and meta-consciousness

The clock task developed by Libet and its later variants are used to obtain subjective reports of the study participants about the onset time of decisions in consciousness. An implicit assumption underlying the conclusions of Libet et al. (1983) and Soon et al. (2008) is that these reported times are unbiased indices of the true onset times of conscious decisions. Is this assumption justified?

The following findings support the validity of the Libet clock for determining subjective event times.

1. Libet verified in his original study that participants were able to report relatively accurately the onset of tones and finger movements (Libet et al., 1983).
2. Reported times of finger movements were found to be relatively robust to manipulations of the physical characteristics of the rotating spot and to different instructions given to the participants (Pockett and Miller, 2007).

However, these validations are based on the timing of *external* events. When it comes to the timing of *internal* intentions, there is accumulating evidence that Libet's clock method is problematic. Hence, there are several other possible interpretations of the delayed reports of conscious decisions which disagree with the conclusion that decision stages before the reported times are unconscious.

**Figure 4:**
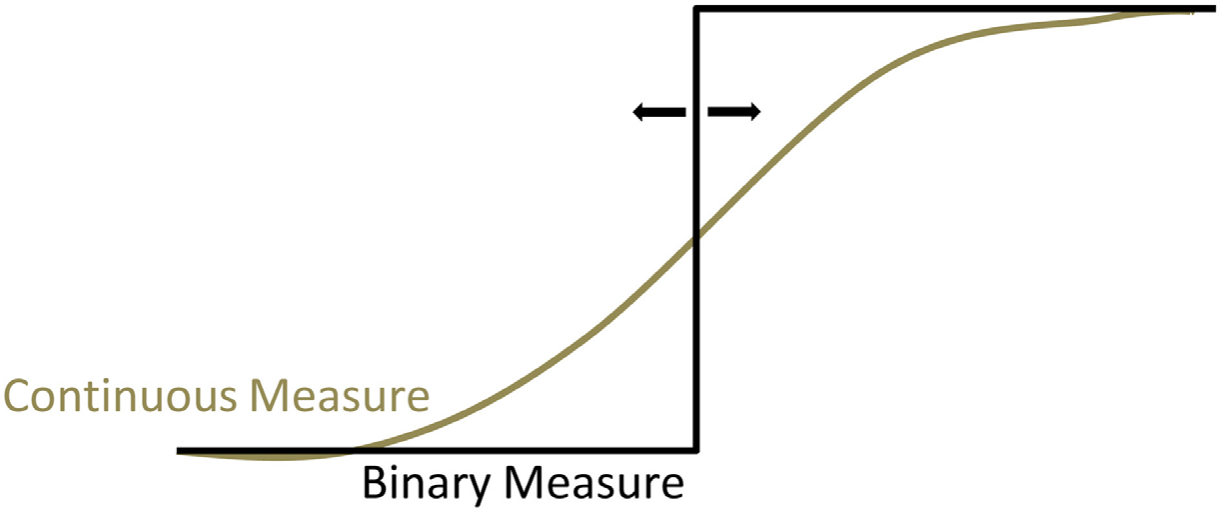
**The Libet clock requires a translation of the continuous experience of consciousness to a binary onset time**. This may introduce delays in the reported intention onset.

1. Subjective onset times of intention onset are influenced by perceptual information obtained during and after the decision. Thus, it is possible to manipulate the reported decision times by presenting a deceptive auditory signal just after the hand movements chosen by the participants (Banks and Isham, 2009; Rigoni et al., 2010), or by transiently disturbing the neural processing just after the movements with a magnetic pulse applied to the SMA (Lau et al., 2007). Similarly, the rotation speed of the clock has significant confounding impact on the intention times reported by the participants (Danquah et al., 2008).
2. Another issue concerns the instructions given to the study participants to report the time when they “make their conscious motor decision” or when they “feel the urge to move.” It is likely that the participants interpreted this as having to indicate the time when a final decision was reached and a corresponding intention made and not as the time when they started *considering* the options. Indeed, as discussed above, the times reported by the participants match the time of increased high-gamma oscillations in brain regions responsible for final intention formation (Guggisberg et al., 2011). This does, however, not necessarily mean that the preceding decision stages from which earlier movement predictors were extracted were unconscious. During decision-making, choices are based on values attributed to the available options (Sugrue et al., 2004; Sanfey et al., 2006). Hence, the option evaluation stages must determine, at least for rational choices, the decision made in subsequent intention stages such that the option with greatest final subjective value is chosen. It is therefore not surprising that one can decode, with a certain probability, future decisions from early neural processes underlying goal evaluation and value representation. Study participants may not have reported their decision at this time simply because it was not final yet, and not because it was unconsciously initiated. If the participants had been instructed to report the earliest time when they consciously start *considering* the options, they would probably have reported much earlier onset times that might even have matched the times obtained with the fMRI classifiers.
3. The subjective timing tasks yield a binary measure of the content of consciousness over time: before the reported onset time, consciousness of decisions is absent; at the reported time it becomes present (Fahle et al., 2011). In contrast, the markers used for assessing the onset of neural processes mediating decisions are continuous measures of voltage (in the case of the BP, LRP, or high-gamma oscillations), spiking rate, or probability (in the case of the fMRI classifiers). However, as shown above, the decision for a movement does not abruptly appear in a binary manner, but is gradually constructed. Hence, the Libet paradigm imposes a translation of the continuous tendency to opt for a movement to a binary measure. Depending on when the participants set the cutoff for this binary translation, this will inevitably lead to a smaller or greater delay in reported onset of consciousness (see Figure 4). Indeed, Fahle et al. (2011) have shown that when participants can use a continuous measure to indicate their tendency for a movement decision such as joystick movements, they report to become conscious of their decisions much earlier than suggested by a binary measure.
4. Let us consider what actually happens in the participants' mind and brain when they introspect the onset time of their conscious decisions. A straightforward answer proposed already by the philosopher Brentano (1874) would be that introspection arises directly and automatically from ongoing conscious processing. Participants perform the tasks of the Libet paradigm with different thoughts entering consciousness and as soon as the content of consciousness is their decision, they can automatically memorize and report it. However, several lines of evidence suggest that it is not as simple as this. The phenomenologist tradition which is particularly devoted to the examination of the mind with introspection has long pointed out that ongoing consciousness is not the same thing as introspection (Husserl, 1984; Gallagher and Zahavi, 2010). Ongoing *first-order consciousness* is defined as the direct, continuous, unreflected experience, whereas *introspection* (also named *meta-consciousness*) is defined as an additional intermittent, explicit re-representation of the contents of first-order consciousness (Schooler, 2002; Marcel, 2003; Overgaard and Sorensen, 2004). Experiencing self-paced movement decisions would be an example of content in first-order consciousness, whereas determining that I have just now made a decision, as it is required in Libet's clock paradigm, is an instance of introspection. More recent research has experimentally corroborated that there are dissociations between ongoing first-order consciousness and intermittent introspection and that introspection can lead to transformations and misrepresentations of the original conscious experience (Schooler, 2002; Marcel, 2003).
  First-order consciousness and introspection also dissociate with regards to the neural structures they rely on. First-order consciousness of the intention to move is associated with an activation of the (pre-)SMA and the posterior parietal cortex (Figure 3A) (Lau et al., 2004; Guggisberg et al., 2011) whereas introspection of intention times with the Libet clock activates additional brain areas: the bilateral angular gyri (Figure 3B) (Guggisberg et al., 2011). Electrical stimulation of the SMA provokes an urge to move a specific body part in a specific direction and even a physical movement with stronger stimulation intensities (Fried et al., 1991). Conversely, electrical stimulation of the angular gyri induces the impression of having the desire to move, which is rather unspecific with regard to which movement is to be performed and not accompanied by the actual movement, even with strong stimulation intensities (Desmurget et al., 2009). The difference between the endogenous aspect of angular stimulations and the obligatory motor preparation character of SMA stimulations fits well with their different implications in introspection vs. first-order consciousness. Furthermore, patients with lesions in the angular gyri are incapable of introspecting the time of their decisions, but they keep their ability to make first-order conscious decisions (Sirigu et al., 2004), thus suggesting that the angular gyri mediate the introspection of decisions, but not the decisions themselves. The SMA may in fact have a double role and additionally contribute to introspective reports of event times. After an early activation peak during intention formation, it is reactivated after action execution (Cunnington et al., 2006; Guggisberg et al., 2008) and this post-action reactivation seems to have a causal role in introspection of event times (Lau et al., 2007). Although different interpretations are possible for each of these findings, they suggest together with behavioral evidence that healthy humans recruit specific introspection networks to access and report relevant neural processes in separate decision-making networks. This means that subjective event times obtained with the Libet clock do not directly reflect the original, first-order conscious experience of the participants (which is mediated by decision-making networks, Figure 3A) but depend on interactions with additional introspection processes (Figure 3B).
  Introspection processes can thereby distort the original conscious experience. Since the neural mechanisms of introspection have resource constraints, they are susceptible to interference with other ongoing neural activity. Thus, introspection may be disturbed by concurring tasks that require neural resources in close temporal proximity to the act of introspection, as it has indeed been observed in numerous studies (Moutoussis and Zeki, 1997; Eagleman and Sejnowski, 2000; Haggard et al., 2002; Stetson et al., 2006; Corallo et al., 2008; Banks and Isham, 2009). The resulting error can be in the order of ~50 ms (Banks and Pockett, 2007). In addition, introspection can misrepresent the original experience for instance when one verbally reports non-verbal or ambiguous experiences (Schooler, 2002; Marcel, 2003).
  Taken together, evidence from several lines of research suggests the existence of two hierarchical levels of consciousness: a first-order consciousness and an intermittent introspective consciousness which is also named meta-consciousness (Schooler, 2002). Subjective reports obtained in the Libet paradigm arise in meta-consciousness (Guggisberg et al., 2011). Hence, even though early decision stages seem to be “meta-unconscious”, i.e., ignored by or inaccessible to introspection/meta-consciousness, they may still be first-order conscious.
  Evidence that this is indeed the case comes from a study which used an alternative method to the Libet clock for determining the onset of conscious movement decisions. Participants were presented tones in random intervals before self-paced movements. Each tone prompted them to decide immediately (rather than using *post-hoc* recall as in the Libet paradigm), whether or not there was an intention to move. The onset of conscious decisions measured with this alternative method turned out to be much earlier than when measured with the Libet clock, i.e., on average 1.4 s before the movements (Matsuhashi and Hallett, 2008).

In sum, several lines of research consistently demonstrate limitations of the clock method used in Libet's paradigm, although the severity of these limitations is still somewhat controversial. In the best case, the onset times of conscious intentions obtained with Libet's clock method are merely imprecise. Alternatively, they may represent meta-conscious inferences rather than the primary conscious experience of intention. In the worst case, humans do not have introspective access to their intentions and merely report *post-hoc* confabulations based on sensory perceptions during and after the action. In any case, the clock method fails to capture early contents of consciousness during goal evaluation. Many of these concerns also apply to an fMRI variant of Libet's clock method (Soon et al., 2008). It is therefore unclear whether participants are aware of early neural processes which are predictive for subsequent decisions.

## Summary and conclusions

The work of Libet has inspired generations of researchers and has started a still ongoing experimental quest on the interaction between neural processing and consciousness. He has been the first to show that decisions are not instantaneous or purely mental events, but that they represent the readout of an implementational brain process. However, findings obtained with Libet's clock paradigm still seem to suggest that consciousness of intentions is a single, instantaneous event. Most current interpretations of the research inspired by Libet assume that unconscious neural decision processes build up until they cross a threshold which then enables the instantaneous appearance of a full-blown conscious intention (Soon et al., 2008; Fried et al., 2011). However, this instantaneous appearance of conscious intentions might be an artifact of the method used for assessing the contents of consciousness. Studies using alternatives to the Libet clock have suggested that intention consciousness is a multistage process just as the neural mechanisms of motor decisions (Matsuhashi and Hallett, 2008; Fahle et al., 2011). The time of conscious intentions reported by the participants therefore might be only the culmination of preceding conscious deliberations, not a unique and instantaneous event. If this is true, the delay between the onset of neural predictors of motor decisions and conscious intentions reported with the Libet clock is not due to unconscious neural processes but due to conscious evaluations which are not final yet. The data currently available does not allow drawing definitive conclusions and other interpretations are equally possible. There are in fact instances when motor decisions are clearly initiated unconsciously (Custers and Aarts, 2010). The question whether early neural predictors of decision outcome are conscious is currently unresolved and requires more refined methods for assessing consciousness. Yet, the model proposed here is consistent with current findings without putting into question our conviction that we can consciously influence motor decisions. Given recent observations that denying the existence of free will has negative behavioral consequences (Vohs and Schooler, 2008; Baumeister et al., 2009; Rigoni et al., 2012, 2013b; Alquist et al., 2013), this new conclusion could have significant behavioral relevance.

## Future directions

Like the assessment of neural activity, the measurement of consciousness turns out to have its pitfalls and to require special care. However, there is no reason to go back to behaviorist reasoning that any scientific study using introspection and subjective reports is futile. On the contrary, future efforts should take into account current knowledge about the hierarchic structure of consciousness and the validity of different methods for measuring consciousness. Now that we have available valid markers of neural processing, we should try to develop and use refined markers also for the assessment of the contents of consciousness. In particular, it seems to be important to minimize the distorting effect of meta-consciousness and *post-hoc* perceptions. Table 1 lists several promising alternatives to Libet's clock task. Although it may be difficult to avoid all meta-conscious inferences, the following strategies have been proposed to favor the apprehension of first-order consciousness.

**Table 1:** **Overview of alternatives to the Libet clock for investigating consciousness during decision-making**.

**Approach**	**References**
*Thinking aloud*: Participants provide concurrent verbal reports about their ongoing conscious considerations	Fox et al. (2011)
*Joystick movements*: Participants indicate their current propensity to move with gradual joystick movements	Fahle et al. (2011)
*Random and unexpected sampling*: A signal instructs participants to report their immediate experience	Hurlburt and Heavey (2004), Matsuhashi and Hallett (2008)

– Use simple instructions and tasks with which the participants have sufficient skills (Marcel, 2003).
– Obtain immediate reports about current intentions rather than *post-hoc* recalls (Matsuhashi and Hallett, 2008).
– Use intermittent and unexpected probing to favor spontaneous and non-reflexive reports (Hurlburt and Heavey, 2004; Matsuhashi and Hallett, 2008).
– Bring the participants in an immersive state of mind allowing them to make more direct and non-reflexive recollections of their first-order consciousness (Marcel, 2003).
– Use continuous rather than binary measures (Fahle et al., 2011).
– Let participants speak aloud their ongoing thoughts about their intentions (Fox et al., 2011).
– Obtain more than one subjective measure of consciousness to be able to estimate the robustness and consistency of the assessment (Marcel, 2003).

### Conflict of interest statement

The authors declare that the research was conducted in the absence of any commercial or financial relationships that could be construed as a potential conflict of interest.
