# Supplementary material for: Timing and awareness of movement decisions: does consciousness really come too late?
Source: Front Hum Neurosci. 2013 Jul 30;7:385. doi: 10.3389/fnhum.2013.00385 (PMC3746176; doi:10.3389/fnhum.2013.00385)
Supplement: Supplementary file 1 [file DataSheet1.docx]

**Supplementary Material to**

Timing and awareness of movement decisions:
Does consciousness really come too late?

*Adrian G. Guggisberg, Anaïs Mottaz*

Division of Neurorehabilitation, Department of Clinical Neurosciences, University Hospital and University of Geneva

**Supplementary Table 1. Overview of neural onset times during motor decisions reported in the literature.** Onset time is relative to button press or electromyogram onset.

| **Neural events** | **Onset Time [ms]** |
| --- | --- |
| *Bereitschaftspotential* |  |
| Deecke et al. ([1969](#_ENREF_17)) | -850 |
| Shibasaki et al. ([1980](#_ENREF_69)) | -1325 |
| Libet et al. ([1982](#_ENREF_50)) |  |
| Type 1 | -1055 |
| Type 2 | -577 |
| Type 3 | -240 |
| Libet et al. ([1983](#_ENREF_49)) |  |
| Type 1 | -1025 |
| Type 2 | -535 |
| Type 3 | -270 |
| Keller and Heckhausen ([1990](#_ENREF_42)) |  |
| Experiment 1 | -462 |
| Experiment 2 | -494 |
| Papa et al. ([1991](#_ENREF_59)) | -1290 |
| Jahanshahi et al. ([1995](#_ENREF_39)) | -1295 |
| Haggard and Eimer ([1999](#_ENREF_35)) | -1750 |
| Trevena and Miller ([2002](#_ENREF_81)) |  |
| Experiment 1 | -850 |
| Experiment 2 | -1900 |
| Sirigu et al. ([2004](#_ENREF_73)) |  |
| W judgments | -1214 |
| M judgments | -974 |
| Matsuhashi et al. ([2008](#_ENREF_53)) | -2170 |
|  |  |
| *Lateralized Readiness Potential* |  |
| Haggard and Eimer ([1999](#_ENREF_35)) | -845 |
| Trevena and Miller ([2002](#_ENREF_81)) |  |
| Experiment 1 | -300 |
| Experiment 2, fixed decisions | -200 |
| Experiment 2, free decisions | -400 |
| Matsuhashi et al. ([2008](#_ENREF_53)) | -590 |
|  |  |
| *fMRI predictors* |  |
| Soon et al. ([2008](#_ENREF_75)) | -7600 |
|  |  |
| *Neuronal firing rate* |  |
| Fried et al. ([2011](#_ENREF_28)) | -1500 |
|  |  |
| *High-gamma power difference in primary motor cortices* | |
| Guggisberg et al. ([2011](#_ENREF_32)) | -194 |

**Supplementary Table 2. Overview of subjective event times during motor decisions reported in the literature.** Onset time is relative to button press or electromyogram onset.

| **Subjective Events** | **Onset Time [ms]** |
| --- | --- |
| *W (Intention, measured with Libet clock)* | |
| Libet et al. ([1983](#_ENREF_49)) | -192 |
| Keller and Heckhausen ([1990](#_ENREF_42)) | -205 |
| Haggard and Eimer ([1999](#_ENREF_35)) |  |
| Fixed decisions | -355 |
| Free decisions | -353 |
| Lau et al. ([2004](#_ENREF_47)) | -228 |
| Sirigu et al. ([2004](#_ENREF_73)) | -239 |
| Lau et al. ([2007](#_ENREF_48)) | -148 |
| Fried et al. ([2011](#_ENREF_28)) | -193 |
| Miller et al. ([2011](#_ENREF_54)) | 30 |
| Guggisberg et al. ([2011](#_ENREF_32)) | -255 |
|  |  |
| *M (Movement, measured with Libet clock)* | |
| Libet et al. ([1983](#_ENREF_49)) | -86 |
| Haggard and Eimer ([1999](#_ENREF_35)) |  |
| Fixed decisions | -89 |
| Free decisions | -98 |
| Lau et al. ([2004](#_ENREF_47)) | -29 |
| Sirigu et al. ([2004](#_ENREF_73)) | 20 |
| Lau et al. ([2007](#_ENREF_48)) | -50 |
| Guggisberg et al. ([2011](#_ENREF_32)) | -54 |
|  |  |
| *T (Intention, measured with random sampling)* | |
| Matsuhashi et al. ([2008](#_ENREF_53)) | -1420 |
|  |  |
| *P (Intended movement cannot be stopped anymore, measured with random sampling)* | |
| Matsuhashi et al. ([2008](#_ENREF_53)) | -130 |
